# Seasonal variation in wing size and shape of *Drosophila melanogaster* reveals rapid adaptation to environmental changes

**DOI:** 10.1038/s41598-022-18891-5

**Published:** 2022-08-26

**Authors:** Banu Şebnem Önder, Cansu Fidan Aksoy

**Affiliations:** Genetic Variation and Adaptation Laboratory, Department of Biology, Faculty of Science, Hacettepe University, Ankara, Turkey

**Keywords:** Genetics, Evolutionary biology, Evolution, Evolutionary genetics

## Abstract

Populations in seasonal fluctuating environments receive multiple environmental cues and must deal with this heterogenic environment to survive and reproduce. An enlarged literature shows that this situation can be resolved through rapid adaptation in *Drosophila melanogaster* populations. Long-term monitoring of a population in its natural habitat and quantitative measurement of its responses to seasonal environmental changes are important for understanding the adaptive response of *D. melanogaster* to temporal variable selection. Here, we use inbred lines of a *D. melanogaster* population collected at monthly intervals between May to October over a temporal scale spanning three consecutive years to understand the variation in wing size and wing shape over these timepoints. The wing size and shape of this population changed significantly between months and a seasonal cycle of this traits is repeated for three years. Our results suggest that the effects of environmental variables that generated variation in body size between populations such as latitudinal clines, are a selective pressure in a different manner in terms of seasonal variation. Temperature related variable have a significant nonlinear relation to this fluctuating pattern in size and shape, whereas precipitation and humidity have a sex-specific effect which is more significant in males.

## Introduction

Environmental variables such as temperature, photoperiod and food availability vary over spatial and temporal scales, driving adaptive divergence among populations, and providing information concerning adaptive evolution. Because of this fact, the ecological and evolutionary impact of latitude^1–4^, altitude^4–9^ and seasons (^10,11^ and references therein^12–14^), on organisms’ life history have long been a topic of interest. Natural populations distributed along latitudinal or altitudinal gradients have been widely used for studies of spatial selection, where the most predictable abiotic factor is the temperature between several climatic and ecological factors. In a similar manner, seasonality leads to predictable changes in a variety of environmental parameters over time, and phenotypic and genetic changes in natural populations among seasons demonstrate evolutionary responses to seasonal fluctuations^15–18^. Thus, the seasonal variation in life histories of organisms is a type of temporal variation^19^.

The annual pattern in photoperiod and temperature varies with latitude and seasonal changes in these environmental parameters can form seasonally consistent patterns, e.g., winter versus summer seasons in temperate regions or wet versus dry seasons in tropical regions. Thus, seasonally fluctuating environments can increase the selective pressures as a function of time, in comparison to stable non-seasonal environments. These variations in the measure of seasonality can also be one of the mechanisms where spatial clines are formed and appear as a result of the seasonal phase cline models^20^. For example, the individuals of a high-latitude *Drosophila melanogaster* population collected in autumn are genetically and phenotypically similar to low-latitude populations^21–23^. The organism’s evolutionary response to seasonality, which is triggered by a complex and fluctuating environmental pressure, ensures that existing genetic polymorphisms are preserved. In multivoltine species, seasonal selective pressures can change across generations; in this case, natural selection can cause cyclical changes in allele frequencies^12–14,24–28^. The first findings of genetic differences related to seasonal variation reported in *Drosophila pseudoobscura*^29^ and *Drosophila funebris*^30^. Many further studies have demonstrated cyclic seasonal changes in the chromosomal inversion frequency in species of *Drosophila,* e.g., *D. robusta*^31^, *D. persimilis*^10^, *D. melanogaster*^32^, *D. subobscura*^33^, *D. mediopunctata*^34^, suggesting the presence of seasonal adaptation.

Accordingly, seasonality is an important selective force of adaptation in a broad range of invertebrate species, however, it is unclear how populations adapt rapidly to seasonal fluctuations. Recent studies of a seasonal genomic oscillations in *Drosophila* populations in North America provides an important insight into rapid adaptation to seasons^12^. Bergland et al.^12^ identified alleles called ‘fall like’ and ‘spring like’, which have the ability to fluctuate rapidly between seasons, which confirms that the populations rapidly adapt to changing environments. Studies investigating phenotypic variation, such as fecundity, developmental time, stress tolerance^22^, and immune response^25^ also show seasonal patterns in common-garden experiments. In the same manner, temperate populations from North America and Europe demonstrate parallel seasonal allele frequency change across the two continents^13^. Together, these results support that seasonal adaptation could be a general phenomenon of temperate *Drosophila* populations^12–14^.

Many organisms exhibit clinal patterns of body size^35–41^ and well-known of these patterns is Bergmann’s rule^35^. According to Bergmann’s rule, close species, or individuals of the same species in endotherms are larger in cold climates or higher latitudes than those in warmer climates or lower latitudes. Since its introduction, the rule’s applicability to invertebrates has always been questionable and gave rise to the thoughts of lack of a general pattern in ectotherms for body size clines, yet the result of more than one mechanism for these taxa.

Since body size is correlated with various environmental factors and therefore a strong adaptive morphological character, it has long been accepted as the most significant feature of organisms. Body size is also highly correlated with many life-history traits^42^ and determines the abundance of species^43^. For example, in *Drosophila* species, body size is correlated with developmental time^44,45^, reproductive success^46–48^ and life span^49–51^. In the light of adaptation, the question that the most researchers are interested in is how and why body size varies through time (i.e., temporal variation) and geography (i.e., spatial variation). In terms of geographic variation, body size increases with latitude in some invertebrate species^52^, but most other invertebrates do not follow this pattern^53^. Consequently, the temporal and spatial variation in body size is especially contentious^54^ because the patterns that we have observed do not have a consensus between ectothermic and endothermic organisms (e.g., between insects and vertebrates).

However, the majority of scientific evidence indicates that temperature is the primary cause for the observed clinal pattern. For example; studies on the thermal adaptation under the laboratory conditions show that body size evolution is concordant with latitudinal sorting in *Drosophila*^55,56^. Many other ecological and environmental parameters, such as rainfall, humidity and UV exposure, vary along latitudes. Recent studies have attempted to identify the ecological and environmental factors that may have resulted in latitudinal clines for body size^40,57,58^. Furthermore, observed latitudinal body size clines in *Drosophila* are suggested to be the direct result of adaptation^59–61^. Common-garden experiments show that clines in body size are continuous, providing a shred of clear evidence that it has a genetic basis^62^.

Adaptation to new environments usually occurs through the adaptation of polygenic characters, as selection alters the optimal values of life history traits in new environment. One of the best studied polygenic morphological characters in *Drosophila* is body size^63,64^ and previous studies showed positive correlation between size related traits such as thorax length, body weight and wing size in (e.g.,^45,65,66^), although wing size is the most commonly used proxy of body size^67^. Moreover, as body size is correlated with many life history traits such as lifespan^49–51^, mating behavior^68^ and flight ability^69^, any of the morphological traits related to body size can be used as a “model trait” to track changes in traits correlated with body size.

In this study, we investigate putative seasonal selection pressures on wing size and shape using inbred lines derived from a temperate *D. melanogaster* population collected at monthly intervals between May to October over a temporal scale spanning three consecutive years from 2014 to 2016. Our findings show a significant effect of seasonality on both morphological traits. The pattern of seasonal variation in wing morphology is consistent across years, implying rapid seasonal adaptation to changing climatic factors across seasons.

## Material and methods

The wild population of *Drosophila melanogaster* was collected from an orchard in Yeşilöz, Turkey (40.30° N, 32.34° E) monthly from June to October in 2014 and May to October in 2015 and 2016. Every collection occurred approximately with a 30-day interval with traps hung always in the same location for 1–2 days. Isofemale lines were established upon collection and were maintained at standard conditions; 25 °C and 60–65% humidity on a 12:12 h light/dark cycle on a standard cornmeal-agar-sugar-yeast medium.

After two generations, inbred lines were established from isofemale lines through 20 generations of full-sib mating which is considered in a theoretically inbreeding level of *f* ≅ 1^70^. A minimum of 10 inbred lines per collection month and year were used for the wing measurements, summing up to a total of 166 inbred lines in this study. One exception to this is June 2014 sampling time-point, which has been phenotype only for 6 lines due to low survival upon inbreeding.

### Wing measurements

To obtain flies for the wing size and shape measurements, we put 25 adult flies of each sex in 3 replicate bottles containing 50 mL of standard medium. To prevent overcrowding the parents were discarded after 3 days from the bottles. Upon eclosion, at least 25 flies of each sex moved into egg-collection chambers with %2 agar medium, and a pile of yeast paste at 25 °C. After 6–8 h, 150 eggs are collected from the surface with fine forceps and transferred evenly to 3 replicate vials containing 7 mL standard medium. The collected eggs are allowed to develop under standard conditions, as described above. Three days after eclosion adult flies from every replicate vial were collected and stored in ethanol. These standard conditions are used for minimizing the environmental effects on body size.

A minimum of 15 individuals per sex have been dissected from 166 inbred lines, summing up to a total of 5377 wings being measured. The left wing was removed with fine forceps and mounted on a microscope slide in Entellan® (Merck Millipore). All wings were photographed by using a camera attached stereo microscope (Leica S9i) and then digitized as jpeg format.

### Analysis

**Figure 1:**
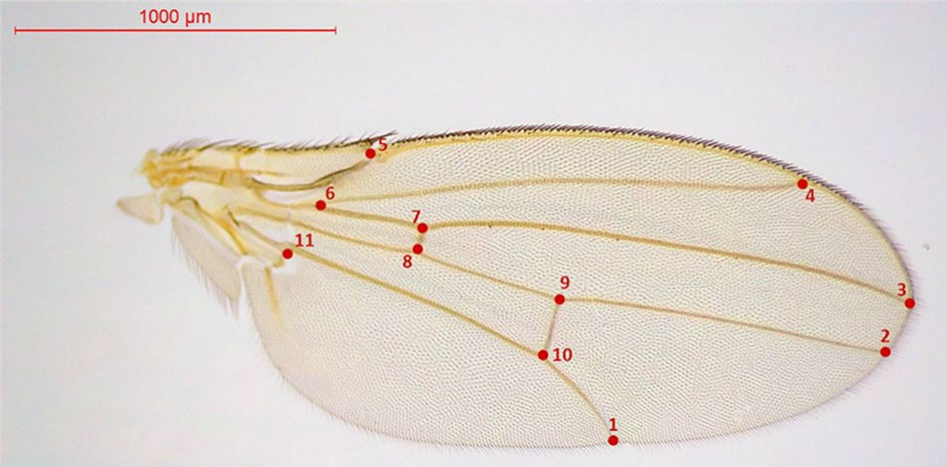
The positions of 11 landmarks (red points) used in shape analysis.

For the estimation of wing shape, 11 landmarks were digitized of the left wing (Fig. 1) of each fly using *tpsUtil* and *tpsDig2*^71,72^. The *tps* files were imported to the MorphoJ software version 1.07a^73^. To remove the position and orientation variations from the coordinates the raw landmark coordinates were aligned and superimposed using a Procrustes Fit function and wing shape variation was analyzed. The effects of sex and sampling months on shape were tested with Procrustes ANOVA. MANOVA was performed on shape coordinates (Procrustes coordinates) to test whether the population diverged in shape by months. To maximize the differences between groups by comparing mean shapes a Canonical variate analysis (CVA)^73^ was used to analyze discrimination between groups where month used as a classifier. The pairwise differences in shape were analyzed by using a permutation test (10.000 rounds) with Procrustes distances, which is the sum of the distance between the landmarks. Additionally, a discriminant function analysis (DFA) was used to find the shape differences between sampling months. To test for allometry (relationship of body size to shape) a multivariate regression of shape (the Procrustes coordinates as dependent variable) on size (centroid size as independent variable) was performed, pooled within sub-groups of lines, with a permutation test with 10,000 rounds for testing statistical significance. All analyses were performed using MorphoJ version 1.07a^73^.

Wing centroid sizes (computed from raw data of landmarks) were used as a measure of wing size. Male and female flies were analyzed separately due to the significant sexual dimorphic differences in body size. The centroid size (CS) data were first analyzed by a Shapiro–Wilk normality test. As the data did not follow a normal distribution, analyses were continued by nonparametric tests. To determine the difference between the three replicate of wing measurements, a Kruskal–Wallis test was performed. Since there was no significant difference between replicas (p > 0.05), the data was pooled. Differences among months of CS were computed with the Kruskal–Wallis test followed by Dunn test with Bonferroni correction for multiple comparisons and pairwise tests using the “ggstatsplot” package^74^. All analyses were performed in *R software*^75^ version 4.1.2.

To test the relationship between climatic variables and centroid size we used climatic variables recorded monthly by the Turkish State Meteorological Service in Güdül, 6 km far from the sampling point (Table 1). Monthly mean temperature (T_mean_), monthly mean maximum temperature (T_max_), monthly mean minimum temperature (T_min_), monthly mean humidity (H_mean_), monthly mean maximum humidity (H_max_), monthly mean minimum humidity (H_min_) and total monthly precipitation (P_total_) variables were used. The analyses were split in two parts: (i) using the climatic data for 15 days before the collection dates, (ii) using the climatic data for the collection days. Our preliminary results supported a strong relationship with climatic variables prior the collection dates and analyses were performed with these variables (given in Table 1).

**Table 1:** Climatic variables for Yeşilöz recorded monthly by the Turkish State Meteorological Service (average values of the month 15 days prior to the collection date).

	T_mean_ (℃)	T_max_ (℃)	T_min_ (℃)	H_mean_ (%)	H_max_ (%)	H_mın_ (%)	P_total_ (mm)
Jun.14	16.4	23.8	9.7	60.9	89.0	30.3	68.5
Jul.14	19.4	27.4	12.5	61.0	89.7	30.4	83.6
Aug.14	25.1	33.7	16.8	42.2	70.4	17.6	9.1
Sep.14	25.6	34.3	17.5	41.6	69.8	16.8	30.0
Oct.14	18.9	26.6	12.4	57.0	85.3	26.9	56.4
May.15	9.0	16	2.1	57.2	87.0	29.0	26.2
Jun.15	16.8	24.5	9.2	57.2	87.9	27.4	66.3
Jul.15	18.2	25.4	12.6	73.8	98.9	38.9	152.6
Aug.15	24.2	32.7	15.4	44.2	75.1	18.7	0.0
Sep.15	24.6	33.2	16.7	47.3	77.2	20.4	21.2
Oct.15	23.0	31.9	14.9	43.4	71.2	18.5	2.7
May.16	13.5	22.0	5.9	57.2	85.8	24.2	29.3
Jun.16	14.6	22.1	8.1	70.6	98.2	34.9	63.7
Jul.16	21.7	30.1	13.0	53.4	89.1	22.8	16.1
Aug.16	24.6	33.5	15.8	42.5	72.8	18.1	2.4
Sep.16	25.7	34.3	17.2	43.3	73.2	17.6	4.8
Oct.16	19.0	27.7	11.4	50.4	80.6	22.8	32.3

We performed a nonparametric regression analysis using Generalized Additive Models (GAMs)^76^ to estimate temporal trends in wing CS and its relationships to climatic variables. GAMs provide a capture of linear and non-linear relationships between variables^77^. Thus, the model allows better prediction of trends in the means of the variables and associated uncertainties. As the climatic categories were collinear, we have adjusted several models with a single independent variable. The dependent variable was CS and independent variables were monthly mean temperature (T_mean_), monthly mean maximum temperature (T_max_), monthly mean minimum temperature (T_min_), monthly mean humidity (H_mean_), monthly mean maximum humidity (H_max_), monthly mean minimum humidity (H_min_) and total monthly precipitation (P_total_). Models were run using “mgcv” package (version 3.6.2)^78^ in R^75^ with residual marginal likelihood (REML) smoothness selection^78,79^. The corresponding R function is *gam(CS* ~ *s(temperature related variable), method* = *'REML', data) and gam(CS* ~ *s(humidity and precipitation related variable), method* = *'REML', data)*. For CS analyses, final model selection was determined by calculating the Akaike Information Criterion (AIC) for all models where the type of smooth terms specify *thin plate regression splines* (default smooth for *s* terms) or *cubic regression splines* (*bs* = *“cs”*).

## Results

### Wing size variation

We studied the effects of the monthly changing environments on wing size and shape in a temperate population from Yeşilöz, Turkey collected over successive years and months. Inbred lines from each collection month were used for size measurements. While size is a sexual dimorphic trait and CS differ significantly between sexes (χ^2^ = 3985.8, p < 0.0001) (Fig. S1), male and females wing measurements were analyzed separately.

**Figure 2:**
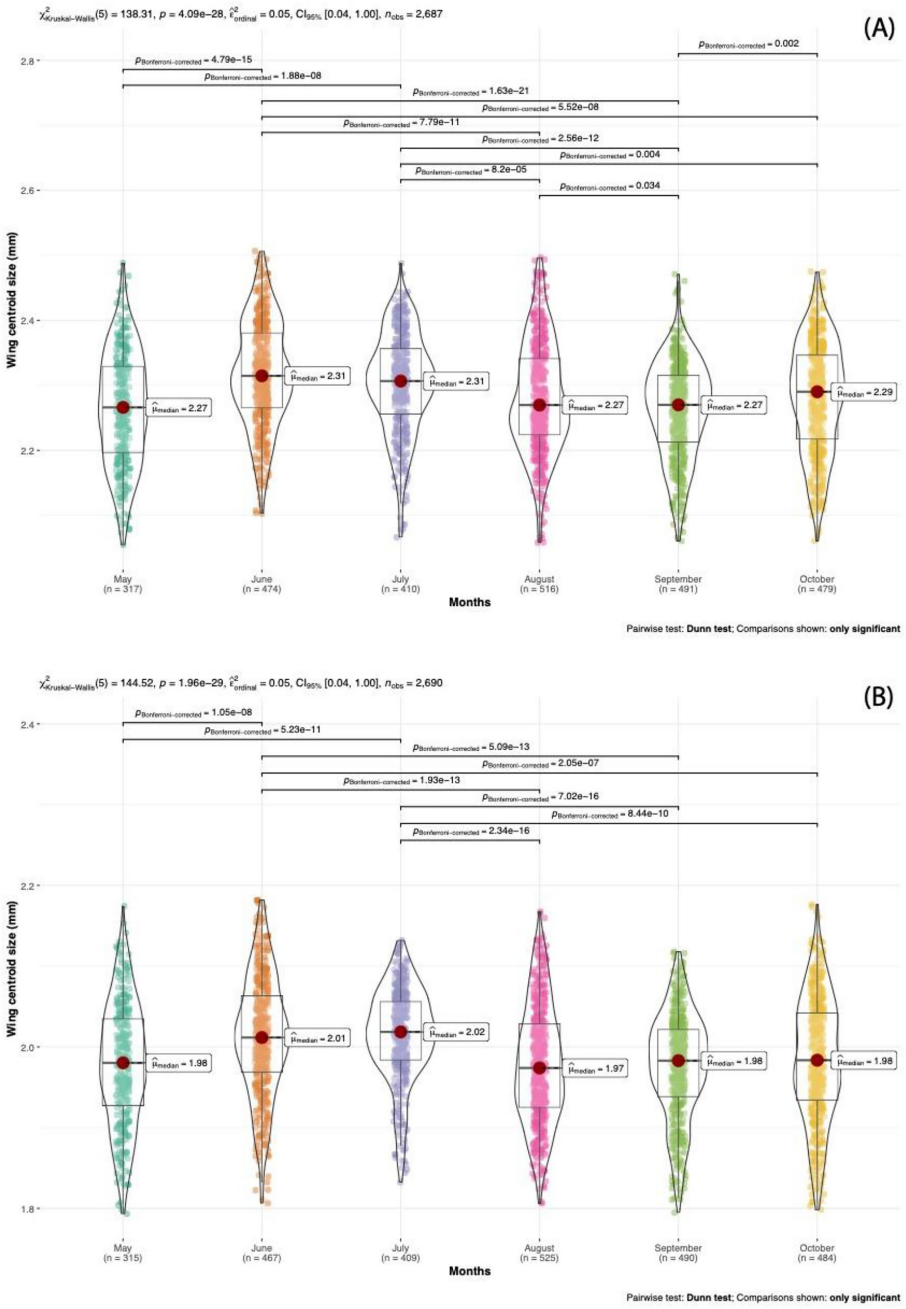
Wing centroid sizes for female (**A**) and male (**B**) by months. Wing centroid size difference among months was analyzed using Kruskal–Wallis test, followed by Dunn post-hoc test with Bonferroni correction.

The mean and standard errors and coefficients of variation for wing measurements for each year and month were given in Table 2 and we found evidence of seasonally fluctuating wing size which is repeated over three years. Mean wing centroid size was smaller in May and rises from June to July, which is followed by smaller sizes in August to September then an increase in October (Table 2, Fig. 2). This fluctuating pattern is repeating every year, except for October 2016, where the wing sizes are decreasing and that was not consistent with the pattern seen in previous years (Fig. 2). Inbred lines originating from June and July are one average bigger than other months in both sexes (Table 2). The coefficients of variation were particularly higher in some months (Table 2). This might be due to a greater genetic heterogeneity between inbred lines in October 2014, June 2015, August 2015 and May 2016.

**Table 2:** Mean values and coefficients of variation (CV’s) for mean wing CS of inbred lines collected from different moths across three years.

	2014				2015				2016			
	n	Mean	SE	CV (%)	n	Mean	SD	CV (%)	n	Mean	SD	CV (%)
**Female**												
May	–	–	–	–	171	2.240	0.00519	3.031	146	2.293	0.0985	4.296
June	175	2.327	0.00569	3.235	159	2.296	0.00704	3.868	140	2.328	0.0760	3.265
July	108	2.310	0.00714	3.216	155	2.300	0.00644	3.487	147	2.299	0.0742	3.227
August	213	2.292	0.00560	3.565	149	2.280	0.00791	4.237	154	2.267	0.0786	3.467
September	181	2.274	0.00532	3.144	159	2.247	0.00570	3.200	151	2.262	0.0793	3.506
October	188	2.296	0.00691	4.128	178	2.294	0.00704	3.068	113	2.236	0.0734	3.282
**Male**												
May	–	–	–	–	174	1.954	0.0596	3.050	141	2.008	0.0834	4.153
June	174	2.023	0.0688	3.402	157	1.992	0.0792	3.975	136	2.023	0.0682	3.371
July	111	2.013	0.0422	2.151	154	2.019	0.0523	2.590	144	2.010	0.0730	3.632
August	212	1.989	0.0715	3.596	152	1.984	0.0737	3.714	161	1.957	0.0712	3.638
September	182	1.979	0.0605	3.058	160	1.959	0.0620	3.165	148	1.988	0.0707	3.556
October	185	1.995	0.0884	4.431	175	1.988	0.0617	3.104	124	1.963	0.0722	3.678

Kruskal–Wallis test exhibited a significance between months in females (χ^2^  = 138.3, df = 5, p-value < 0.0001, Fig. 2) as also in males (χ^2^ = 144.5, df = 5, p-value < 0.0001, Fig. 2). Pairwise comparisons performed with Dunn test showed a significant difference between months except May–August, May–September, May–October, June-July and August-October in females. Whereas pairwise comparisons between months in males shows that only June and July differ significantly (p < 0.0001) from other months, all other comparisons are not significant (Fig. 2).

### Wing shape variation

We digitized 11 landmarks to evaluate differences in wing shape between months. Procrustes ANOVAs detected significant differences in shape among sex (F = 466, 17, df = 18, p < 0.0001) and months (F = 20.99, df = 90, p < 0.0001). The MANOVA on Procrustes coordinates was found to be highly significant, suggesting that wing shape varies significantly among months in females (F = 11.59, df = 90, p < 0.0001) and males (F = 12.08, df = 90, p < 0.0001).

The canonical variate analysis provided a clear discrimination of the months defined by the two first canonical axes which respectively accounted for 38.97% and 26.20% of the among-month variance in females (total 65.17%) and 35.40% and 25.36% of the among-month variance in males (total 60.76%). The results of the canonical variate analysis with Mahalanobis distance and Procrustes distances showed morphological variability across the months (Table S1). Permutation tests (10,000 rounds) for Procrustes distances and Mahalanobis distances among months were significant in all comparisons (Table S1) where May and October lines exhibited difference less than others. The highest Procrustes distance in shapes were found among May and July, where the lowest distance was found between May and October in both sexes. Furthermore, there was a significant difference between sexes (Mahalanobis distance among groups = 2.1648, p < 0.0001) within the population (Figs. S2, S3). The multivariate regression coefficient showed that the effect of size (centroid size) on shape (Procrustes coordinates) was highly significant (P < 0.0001). In addition, we observed a moderate amount of allometry that accounted for 22.06% of the total shape variance. In other words, line specific shape variation explained by size differences.

### Climatic effects for size and shape of wing

To find the environmental factor that led to this repetitive pattern across years we applied a Generalized Additive Model (GAM) analysis to assess the relationship between wing centroid size and the climatic variables.

To estimate the relationship between the dependent and independent variables, the GAM analysis was used. The findings of these relationships were estimated as linear and nonlinear effects using smoothing splines (Fig. S4), results presented in Table 3. Results in Table 3 showed that some models resulted with the effective degrees of freedom (*edf*) values near to 1, the measures suggest a linear pattern. Whereas *edf* values were larger than 1 suggested a nonlinear pattern in the relationship between the dependent and independent variable. Furthermore, the model output has indicated that all spline terms of climate variables were significant variables of wing size (Table 3).

**Table 3:** Results of a GAM model, with the effective degrees of freedom (edf), *F statistics*, and P value using non-parametric smoothers.

Smooth effects of variable	Sex	edf	F statistics	P value
Mean temperature	Female	2.634	2.724	0.0381*
	Male	2.510	2.866	0.0361*
Maximum temperature	Female	2.625	3.016	0.0325*
	Male	2.504	3.122	0.0277*
Minimum temperature	Female	2.565	2.927	0.0347*
	Male	2.475	2.792	0.0417*
Mean humidity	Female	1	6.631	0.0109*
	Male	1	9.409	0.00252**
Minimum humidity	Female	1	5.762	0.0175*
	Male	1	7.875	0.00562**
Maximum humidity	Female	2.627	3.177	0.0208*
	Male	1.752	4.527	0.00961**
Mean precipitation	Female	2.827	3.268	0.0181*
	Male	1	7.548	0.00667**

Temperature related variables have a nonlinear relationship with wing size. Temperature related variables fit well with a cubic regression spline (model selection made by AIC criterion). On the other hand, humidity has a linear effect (edf = 1) on male and females in terms of both mean and minimum values. However, precipitation has a nonlinear effect in females, but a linear effect in males (Table 3).

## Discussion

Seasonal fluctuations are main sources for rapidly changing environments in temperate regions and recent studies provided evidence for rapid change in SNP frequencies among seasons^12–14^. Previous studies also revealed that seasonal variation in adaptive phenotypic traits like thermal and starvation tolerance^22^, and innate immunity^25^ in *D. melanogaster* populations and all together suggesting that many loci respond to seasonal selection. This evidence raises new questions about seasonal adaptation and adaptive phenotypic traits. This is main reason to carry out this study to investigate the presence of seasonal variation in wing size and shape. We have investigated the seasonal variation in wing size and shape in a temperate *Drosophila* population. Our results show that wing size and shape changed significantly between months and the observed seasonal cycle is repeated for three consecutive years. We show that temporal selection acts along seasons in the Yeşilöz population of *D. melanogaster* and wing size and shape exhibit a rapid response among months. Our analysis reveals evidence for temporal varying selection on wing size and shape; and this suggesting differences within population between month from this geographic region.

Body size is characterized by a complex genetic nature, that a large number of genes are involved, and the alleles have small effects of this trait. It is hard to find the adaptive polymorphism for such traits to towards more simple traits with alleles of large effects. Furthermore, the methodology used in this study provides measurements of inbred lines derived monthly from the population, reflecting a random genotypic sample in the population. Our results of the panel of inbred lines support that the source of seasonal variation in wing size and shape is an outcome of rapid seasonal adaptation, as the frequency of some alleles of different loci vary between months and contribute to the variation of size. It is likely that many ecological and environmental variables that changed between months are interacted to generate variation in body size and strong selection occurs between months for this morphological trait.

The wing size and shape variations depend on the amount of genetic variation in the population. The fluctuating response to selection shows that the genetic variation is maintained in the population but allele frequency for loci responsible for size and shape are changed rapidly through seasons. This might partially explain the difference within months and between inbred lines. For example, some individuals of an inbred line might have outliers within a month, however a general pattern persists. Previous studies showed that SNP frequencies in *D. melanogaster* populations vary seasonally^12–14^ and transposable elements (TEs) were significantly associated with seasons^80^. Thus, it is possible that selection favors size and / or size related traits depended on changing environmental variables as our wing size and shape results support.

Temperature has also direct environmental effects on body size in *Drosophila* and other ectotherms and it is known as the major driver for body size clines^35,59,60,62,81–85^. Our results indicated that wing size and shape significantly affected by monthly temperature, humidity and precipitation. Accelerated precipitation and humidity have a significant linear relationship with increased wing size especially on males (Table 3). In contrast to this, temperature has a significant non-linear relationship with wing size (Table 3). Our results show that seasonally varying precipitation and humidity have a higher selective pressure on body size than temperature. Increased humidity and precipitation promote larger body size significantly (Table 3, Fig. S4). Our results are not consistent with some other studies about the relationship between size and precipitation and humidity. For example, the wing size of *Bactrocera tryoni,* an Australian endemic horticultural pest species, is also affected by precipitation but shows a negative relationship with precipitation seasonality^86^. Stilwell et al.^40^ proposed that the latitudinal cline in body size of the seed—feeding beetle *Stator limbatus* generated by humidity and seasonality then temperature, and the results support humidity for a better candidate to explain the body size clines where body size increased with decreased humidity. In a similar manner Gibb et al.^87^ showed that the body size declines as precipitation increases in ant species. Experimental evolution at low and high humidity showed in larger wing areas at low humidity conditions in *D. melanogaster*^88^. For *D. simulans* and *D. mercatorum*, Przybylska et al.^89^ found that the flies were larger in the dry season. Generally, the relationship between humidity and body size is explained by desiccation tolerance. Larger body size decreased the surface-to-volume ratio, and this is correlated with increased water content^90^. Selection for increased desiccation tolerance showed increased wet weight and lipid content in *D. melanogaster*^91^. Such proving’s support the relationship between size and desiccation tolerance moreover humidity. However, our results are inconsistent with this pattern. The study with field collected *Anopheles albimanus* populations have exhibited a positive correlation between wing size and relative humidity^92^ which is consistent with our findings. The inconsistency among the studies with humidity and body size point out the need for further work to understand the relationship between humidity and also precipitation and body size. It is known that humidity has a large impact of some traits in *D. melanogaster*^93–95^ and that rainfall is associated with adaptation in *D. melanogaster* natural populations^96^. Moreover, our results reveal that humidity and precipitation have a greater impact on males than females where temperature selection acts the same direction in both sexes. This sex-specific effect for precipitation and humidity could be explained by the selection in nature on body size in the context of fitness advantage. Body size is a sexual dimorphic trait but the genetic basis of body size is shared in both sexes. But it is known that these genes have diverge effects by sex on body size in *D. melanogaster*^97,98^. Like body size, lifespan and ageing share also a same genetic basis in both sexes but sex-specific effects of lifespan were recorded for *D. melanogaster*^99^ and *D. simulans*^100^. Archer et al.^100^ demonstrated that the sexual and natural selection have sex-specific effects in *D. simulans* where males evolve grater baseline mortality than females under high temperature conditions^100^. Natural selection may be a determinant sex differences for wing size in Yeşilöz population where precipitation and humidity favors larger size in males may due the differences in fitness outcomes.

Populations in seasonal fluctuating environments receive multiple environmental cues and must deal with this heterogeneous environment to survive and reproduce. A growing body of literature shows that this situation is resolved through rapid adaptation in *D. melanogaster* populations. This study shows that body size, an important fitness component, is one of the changing phenotypic traits across seasons. Our results suggest that the effects of environmental variables that generated variation in body size among populations such as latitudinal clines, act as a selective pressure in a different manner in seasonal variation within population. Long-term monitoring of a population in its natural habitat and quantitative measurement of its responses to seasonal environmental changes are important for understanding the adaptive response of population to temporal selection. Future studies should focus on the sex-specific effects of environmental variables to better understand the impact of the environment on the evolution of differences in sexual traits.

## Supplementary Information

Supplementary Information.

## Data Availability

The datasets used and/or analyzed during the current study available from the corresponding author on reasonable request.

## References

[1] Roff D (1980). Optimizing development time in a seasonal environment: the ‘ups and downs’ of clinal variation. Oecologia.

[2] Mittelbach GG (2007). Evolution and the latitudinal diversity gradient: Speciation, extinction and biogeography. Ecol. Lett..

[3] Kapun M, Fabian DK, Goudet J, Flatt T (2016). Genomic evidence for adaptive inversion clines in *Drosophila melanogaster*. Mol. Biol. Evol..

[4] Rajpurohit S, Zhao X, Schmidt PS (2017). A resource on latitudinal and altitudinal clines of ecologically relevant phenotypes of the Indian *Drosophila*. Sci. Data.

[5] Hawkins B, DeVries PJ (1996). Altitudinal gradients in the body sizes of Costa Rican butterflies. Acta Oecol..

[6] Sørensen JG, Norry FM, Scannapieco AC, Loeschcke V (2005). Altitudinal variation for stress resistance traits and thermal adaptation in adult *Drosophila buzzatii* from the New World. J. Evol. Biol..

[7] Pitchers W, Pool JE, Dworkin I (2013). Altitudinal clinal variation in wing size and shape in African *Drosophila melanogaster*: one cline or many?. Evolution.

[8] Klepsatel P, Gáliková M, Huber CD, Flatt T (2014). Similarities and differences in altitudinal versus latitudinal variation for morphological traits in *Drosophila melanogaster*. Evolution.

[9] Ayhan N, Güler P, Onder BS (2016). Altitudinal variation in lifespan of *Drosophila melanogaster* populations from the Firtina Valley, northeastern Turkey. J. Therm. Biol..

[10] Dobzhansky T, Ayala FJ (1973). Temporal frequency changes of enzyme and chromosomal polymorphisms in natural populations of *Drosophila*. Proc. Natl. Acad. Sci. USA.

[11] Williams CM (2017). Understanding evolutionary impacts of seasonality: An introduction to the symposium. Integr. Comp. Biol..

[12] Bergland AO, Behrman EL, O'Brien KR, Schmidt PS, Petrov DA (2014). Genomic evidence of rapid and stable adaptive oscillations over seasonal time scales in *Drosophila*. PLoS Genet..

[13] Machado HE (2021). Broad geographic sampling reveals the shared basis and environmental correlates of seasonal adaptation in *Drosophila*. Elife.

[14] Rodrigues MF, Vibranovski MD, Cogni R (2021). Clinal and seasonal changes are correlated in *Drosophila melanogaster* natural populations. Evolution.

[15] Hairston NG, Dillon TA (1990). Fluctuating selection and response in a population of freshwater copepods. Evolution.

[16] Grant PR, Grant BR (2002). Unpredictable evolution in a 30-year study of Darwin's finches. Science.

[17] Brown CR, Brown MB, Roche EA (2013). Fluctuating viability selection on morphology of cliff swallows is driven by climate. J. Evol. Biol..

[18] Bergland AO, Tobler R, González J, Schmidt P, Petrov D (2016). Secondary contact and local adaptation contribute to genome-wide patterns of clinal variation in *Drosophila melanogaster*. Mol. Ecol..

[19] Haldane JBS, Jayakar SD (1963). Polymorphism due to selection of varying direction. J. Genet..

[20] Rhomberg LR, Singh RS (1986). Evidence for a link between local and seasonal cycles in gene frequencies and latitudinal gene clines in a cyclic parthenogen. Genetica.

[21] Cogni R (2014). The intensity of selection acting on the couch potato gene—spatial–temporal variation in a diapause cline. Evolution.

[22] Behrman, E. L., Watson, S. S., O'brien, K. R., Heschel, M. S., & Schmidt, P. S. Seasonal variation in life history traits in two *Drosophila* species. *J. Evol. Biol.* **28**(9), 1691–1704 (2015). 10.1111/jeb.12690 PMC5089932 26174167

[23] Cogni R (2015). Variation in *Drosophila melanogaster* central metabolic genes appears driven by natural selection both within and between populations. Proc. Biol. Sci..

[24] Wittmann MJ, Bergland AO, Feldman MW, Schmidt PS, Petrov DA (2017). Seasonally fluctuating selection can maintain polymorphism at many loci via segregation lift. Proc. Natl. Acad. Sci. USA.

[25] Behrman, E. L., et al. Rapid seasonal evolution in innate immunity of wild *Drosophila melanogaster*. *P. Roy. Soc. B Biol. Sci.* **285**(1870), 20172599 (2018). 10.1098/rspb.2017.2599 PMC5784205 29321302

[26] Rudman SM (2019). Microbiome composition shapes rapid genomic adaptation of *Drosophila melanogaster*. Proc. Natl. Acad. Sci. USA.

[27] Dowle EJ (2020). Genome-wide variation and transcriptional changes in diverse developmental processes underlie the rapid evolution of seasonal adaptation. Proc. Natl. Acad. Sci. USA.

[28] Garcia‐Elfring, et al. Using seasonal genomic changes to understand historical adaptation to new environments: Parallel selection on stickleback in highly‐variable estuaries. *Mol. Ecol.* **30**(9), 2054–2064 (2021). 10.1111/mec.15879 33713378

[29] Dobzhansky, T. Genetics of natural populations IX. Temporal changes in the composition of populations of *Drosophila pseudoobscura*. *Genetics* **28**(2), 162 (1943). 10.1093/genetics/28.2.162 PMC1209199 17247077

[30] Dubinin NP, Tiniakov GG (1945). Seasonal cycles and the concentration of inversions in populations of *Drosophila funebris*. Am. Nat..

[31] Stalker HD, Carson HL (1949). Seasonal variation in the morphology of *Drosophila robusta* Sturtevant. Evolution.

[32] Stalker, H. D. Chromosome studies in wild populations of *Drosophila melanogaster*. II. Relationship of inversion frequencies to latitude, season, wing-loading and flight activity. *Genetics* **95**(1), 211–223 (1980). 10.1093/genetics/95.1.211 PMC1214217 17249033

[33] Rodriguez-Trelles F, Alvarez G, Zapata C (1996). Time-series analysis of seasonal changes of the O inversion polymorphism of *Drosophila subobscura*. Genetics.

[34] Ananina G (2004). Chromosomal inversion polymorphism in *Drosophila mediopunctata*: seasonal, altitudinal, and latitudinal variation. Genet. Mol. Biol..

[35] Bergmann K (1847). Über die Verhältnisse der Wärmeökonomie der Thiere zu ihrer Größe. Gottinger Studien.

[36] Graves GR (1991). Bergmann's rule near the equator: latitudinal clines in body size of an Andean passerine bird. Proc. Natl. Acad. Sci. USA.

[37] Partridge L, Coyne JA (1997). Bergmann's rule in ectotherms: Is it adaptive?. Evolution.

[38] Ashton KG (2002). Patterns of within-species body size variation of birds: strong evidence for Bergmann's rule. Global Ecol. Biogeogr..

[39] Hallas R, Schiffer M, Hoffmann AA (2002). Clinal variation in *Drosophila serrata* for stress resistance and body size. Genet. Res..

[40] Stillwell RC, Morse GE, Fox CW (2007). Geographic variation in body size and sexual size dimorphism of a seed-feeding beetle. Am. Nat..

[41] Clauss M, Dittmann MT, Müller DW, Meloro C, Codron D (2013). Bergmann′ s rule in mammals: A cross-species interspecific pattern. Oikos.

[42] Stearns SC (1992). The evolution of life histories.

[43] Blackburn TM, Gaston KJ (2001). Linking patterns in macroecology. J. Anim. Ecol..

[44] Robertson, F. W. The ecological genetics of growth in *Drosophila* 6. The genetic correlation between the duration of the larval period and body size in relation to larval diet. *Genet. Res.* **4**(1), 74–92 (1963).

[45] Partridge L, Langelan R, Fowler K, Zwaan B, French V (1999). Correlated responses to selection on body size in *Drosophila melanogaster*. Genet. Res..

[46] Partridge L, Farquhar M (1983). Lifetime mating success of male fruitflies (*Drosophila melanogaster*) is related to their size. Anim. Behav..

[47] Lefranc A, Bundgaard J (2000). The influence of male and female body size on copulation duration and fecundity in *Drosophila melanogaster*. Hereditas.

[48] Long TA, Pischedda A, Stewart AD, Rice WR (2009). A cost of sexual attractiveness to high-fitness females. PLoS Biol..

[49] Partridge L, Fowler K (1992). Direct and correlated responses to selection on age at reproduction in *Drosophila melanogaster*. Evolution.

[50] Rodriguez C, Fanara JJ, Hasson E (1999). Inversion polymorphism, longevity, and body size in a natural population of *Drosophila buzzatii*. Evolution.

[51] Norry FM, Loeschcke V (2002). Temperature-induced shifts in associations of longevity with body size in *Drosophila melanogaster*. Evolution.

[52] Karan D, Dubey S, Moreteau B, Parkash R, David JR (2000). Geographical clines for quantitative traits in natural populations of a tropical Drosophilid: *Zaprionus indianus*. Genetica.

[53] Shelomi M (2012). Where are we now? Bergmann’s rule sensu lato in insects. Am. Nat..

[54] Blackburn TM, Gaston KJ, Loder N (1999). Geographic gradients in body size: a clarification of Bergmann's rule. Divers. Distrib..

[55] Cavicchi, S., Guerra, D., Natali, V., Pezzoli, C., & Giorgi, G. Temperature‐related divergence in experimental populations of *Drosophila melanogaster*. II. Correlation between fitness and body dimensions. *J. Evol. Biol.* **2**(4)**,** 235–251 (1989).

[56] Partridge L, Barrie B, Fowler K, French V (1994). Evolution and development of body size and cell size in *Drosophila melanogaster* in response to temperature. Evolution.

[57] Jones J (2005). Multiple selection pressures generate adherence to Bergmann's rule in a Neotropical migratory songbird. J. Biogeogr..

[58] Stillwell RC, Moya-Laraño J, Fox CW (2008). Selection does not favor larger body size at lower temperature in a seed-feeding beetle. Evolution.

[59] Imasheva AG, Bubli OA, Lazebny OE (1994). Variation in wing length in Eurasian natural populations of *Drosophila melanogaster*. Heredity.

[60] van’t Land, J., P. van Putten, H. Villarroel, A. Kamping & W. van Delden Latitudinal variation in wing length and allele frequencies for Adh and α-Gpdh in populations of *Drosophila melanogaster* from Ecuador and Chile. *Dros. Info. Serv.* **76**, 156 (1995).

[61] Loeschcke, V., Bundgaard, J., & Barker, J. S. F. Variation in body size and life history traits in *Drosophila aldrichi* and *D. buzzatii* from a latitudinal cline in eastern Australia. *Heredity* **85**(5), 423–433 (2000). 10.1046/j.1365-2540.2000.00766.x 11122420

[62] Gilchrist AS, Partridge L (1999). A comparison of the genetic basis of wing size divergence in three parallel body size clines of *Drosophila melanogaster*. Genetics.

[63] Turner TL, Stewart AD, Fields AT, Rice WR, Tarone AM (2011). Population-based resequencing of experimentally evolved populations reveals the genetic basis of body size variation in *Drosophila melanogaster*. PLoS Genet..

[64] Pitchers W (2019). A multivariate genome-wide association study of wing shape in *Drosophila melanogaster*. Genetics.

[65] Reeve ECR (1950). Genetical aspects of size allometry. P. Roy. Soc. B-Biol. Sci..

[66] Cowley DE, Atchley WR (1990). Development and quantitative genetics of correlation structure among body parts of *Drosophila melanogaster*. Am. Nat..

[67] Reeve ECR, Robertson FW (1953). Studies in quantitative inheritance. J. Genet..

[68] Menezes BF, Vigoder FM, Peixoto AA, Varaldi J, Bitner-Mathé BC (2013). The influence of male wing shape on mating success in *Drosophila melanogaster*. Anim. Behav..

[69] Ray RP, Nakata T, Henningsson P, Bomphrey RJ (2016). Enhanced flight performance by genetic manipulation of wing shape in *Drosophila*. Nat. Commun..

[70] Falconer D.S., & Mackay T.F.C. *Introduction to Quantitative Genetics*. (4th ed Benjamin Cummings, Longmans Green: Harlow, UK. 1996).

[71] Rohlf FJ (2001). Comparative methods for the analysis of continuous variables: geometric interpretations. Evolution.

[72] Rohlf, F. J. The tps series of software. *Hystrix* **26**(1), (2015).

[73] Klingenberg CP (2011). MorphoJ: an integrated software package for geometric morphometrics. Mol. Ecol. Resour..

[74] Patil I (2021). Visualizations with statistical details: The 'ggstatsplot' approach. J. Open Source Softw..

[75] R Core Team. *R: A language and environment for statistical computing.* (R Foundation for Statistical Computing, Vienna, Austria 2021).

[76] Wood SN, Pya N, Säfken B (2016). Smoothing parameter and model selection for general smooth models. J. Am. Stat. Assoc..

[77] Pedersen EJ, Miller DL, Simpson GL, Ross N (2019). Hierarchical generalized additive models in ecology: An introduction with mgcv. PeerJ.

[78] Wood SN (2017). Generalized additive models: an introduction with R.

[79] Wood SN (2011). Fast stable restricted maximum likelihood and marginal likelihood estimation of semiparametric generalized linear models. J. R. Stat. Soc. B.

[80] Kapun M (2020). Genomic analysis of European *Drosophila melanogaster* populations reveals longitudinal structure, continent-wide selection, and previously unknown DNA viruses. Mol. Biol. Evol..

[81] David J, Bocquet C, De Scheemaeker-Louis M (1977). Genetic latitudinal adaptation of *Drosophila melanogaster*: new discriminative biometrical traits between European and equatorial African populations. Genet. Res..

[82] Coyne JA, Beecham E (1987). Heritability of two morphological characters within and among natural populations of *Drosophila melanogaster*. Genetics.

[83] Capy, P., Pla, E., & David, J. R. Phenotypic and genetic variability of morphometrical traits in natural populations of *Drosophila melanogaster* and *D. simulans*. I. Geographic variations. *Genet. Sel. Evol.* **25**(6), 517–536 (1993).

[84] James AC, Azevedo RB, Partridge L (1995). Cellular basis and developmental timing in a size cline of *Drosophila melanogaster*. Genetics.

[85] Flatt T (2020). Life-history evolution and the genetics of fitness components in *Drosophila melanogaster*. Genetics.

[86] Zhou Y, Rodriguez J, Fisher N, Catullo RA (2020). Ecological drivers and sex-based variation in body size and shape in the Queensland fruit fly, *Bactrocera tryoni* (Diptera: Tephritidae). Insects.

[87] Gibb H (2018). Habitat disturbance selects against both small and large species across varying climates. Ecography.

[88] Kennington WJ, Killeen JR, Goldstein DB, Partridge L (2003). Rapid laboratory evolution of adult wing area in *Drosophila melanogaster* in response to humidity. Evolution.

[89] Przybylska MS, Roque F, Tidon R (2014). Drosophilid species (Diptera) in the Brazilian Savanna are larger in the dry season. Ann. Entomol. Soc. Am..

[90] Chown SL, Gaston KJ (1999). Exploring links between physiology and ecology at macro-scales: The role of respiratory metabolism in insects. Biol. Rev..

[91] Telonis-Scott M, Guthridge KM, Hoffmann AA (2006). A new set of laboratory-selected *Drosophila melanogaster* lines for the analysis of desiccation resistance: response to selection, physiology and correlated responses. J. Exp. Biol..

[92] Gómez GF, Márquez EJ, Gutiérrez LA, Conn JE, Correa MM (2014). Geometric morphometric analysis of Colombian *Anopheles albimanus* (Diptera: Culicidae) reveals significant effect of environmental factors on wing traits and presence of a metapopulation. Acta Trop..

[93] P. M., Hutchinson, E. W., MacKinley, M. D., & Rose, M. R, Service (1985). Resistance to environmental stress in *Drosophila melanogaster* selected for postponed senescence. Physiol. Zool..

[94] Al-Saffar ZY, Grainger JNR, Aldrich J (1996). Temperature and humidity affecting development, survival and weight loss of the pupal stage of *Drosophila melanogaster*, and the influence of alternating temperature on the larvae. J. Therm. Biol..

[95] Aggarwal DD (2013). Rapid effects of humidity acclimation on stress resistance in *Drosophila melanogaster*. Comp. Biochem. Phys. A.

[96] Bogaerts-Márquez M, Guirao-Rico S, Gautier M, González J (2021). Temperature, rainfall and wind variables underlie environmental adaptation in natural populations of *Drosophila melanogaster*. Mol. Ecol..

[97] Carreira VP, Mensch J, Fanara JJ (2009). Body size in *Drosophila*: genetic architecture, allometries and sexual dimorphism. Heredity.

[98] Carreira VP, Soto IM, Mensch J, Fanara JJ (2011). Genetic basis of wing morphogenesis in *Drosophila*: Sexual dimorphism and non-allometric effects of shape variation. BMC Dev. Biol..

[99] Parker, G. A. *et al.* Genetic basis of increased lifespan and postponed senescence in *Drosophila melanogaster*. *G3 - Genes Genom. Genet.* **10**(3), 1087–1098 (2020). 10.1534/g3.120.401041 PMC7056975 31969430

[100] Archer CR (2015). Sex-specific effects of natural and sexual selection on the evolution of life span and ageing in *Drosophila simulans*. Funct. Ecol..

