# Supplementary material for: Seasonal variation in wing size and shape of Drosophila melanogaster reveals rapid adaptation to environmental changes
Source: Sci Rep. 2022 Aug 26;12:14622. doi: 10.1038/s41598-022-18891-5 (PMC9418266; doi:10.1038/s41598-022-18891-5)
Supplement: Supplementary file 1 — Supplementary Information. [file 41598_2022_18891_MOESM1_ESM.docx]

**Figure S1.** Wing centroid sizes by sex. Wing centroid size difference between sexes was analyzed using chi-square.

**Table S1.** Mahalanobis distances (narrow) and Procrustes distances (bold) derived from canonical variate analysis (CVA) of the wing shape calculated by 10,000 random permutations per test to determine statistical significance. P-values of all months were highly statistically significant (permutation 10,000 rounds in MorphoJ: P < 0.0001).

| FEMALE | May | June | July | August | September | October |
| --- | --- | --- | --- | --- | --- | --- |
| May |  | **0.0062** | **0.0089** | **0.0074** | **0.0073** | **0.0037** |
| June | 0.8891 |  | **0.0049** | **0.0055** | **0.0056** | **0.0048** |
| July | 1.3251 | 0.8420 |  | **0.0051** | **0.0087** | **0.0075** |
| August | 1.1043 | 0.8025 | 0.8801 |  | **0.0072** | **0.0069** |
| September | 1.0152 | 0.7165 | 1.1591 | 0.8904 |  | **0.0058** |
| October | 0.8095 | 0.8646 | 1.0901 | 0.9684 | 0.9862 |  |
| MALE | **May** | **June** | **July** | **August** | **September** | **October** |
| May |  | **0.0070** | **0.0093** | **0.0067** | **0.0070** | **0.0043** |
| June | 0.9043 |  | **0.0053** | **0.0056** | **0.0061** | **0.0046** |
| July | 1.2093 | 0.8519 |  | **0.0060** | **0.0085** | **0.0075** |
| August | 1.0050 | 0.8437 | 0.9095 |  | **0.0065** | **0.0063** |
| September | 0.9869 | 0.8320 | 1.1496 | 0.9054 |  | **0.0052** |
| October | 0.7184 | 0.7000 | 1.0020 | 0.8960 | 0.8844 |  |


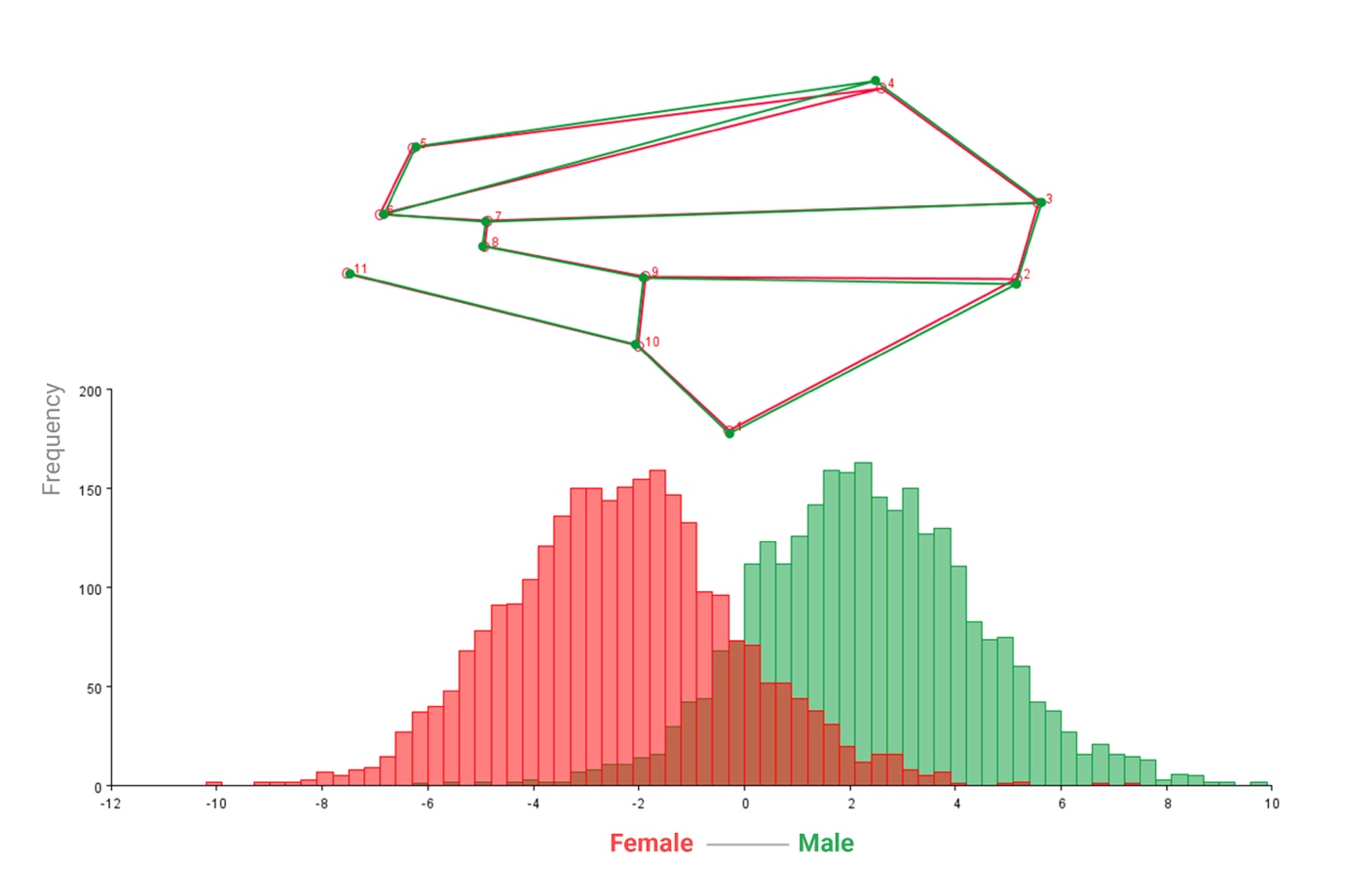


Figure S2. Discriminant function analysis (DFA) (A) Female (red color) vs. male (green color) scores. (B) Wireframe graphs from the canonical variate analysis (CVA) of the female (red) and male (green) groups.


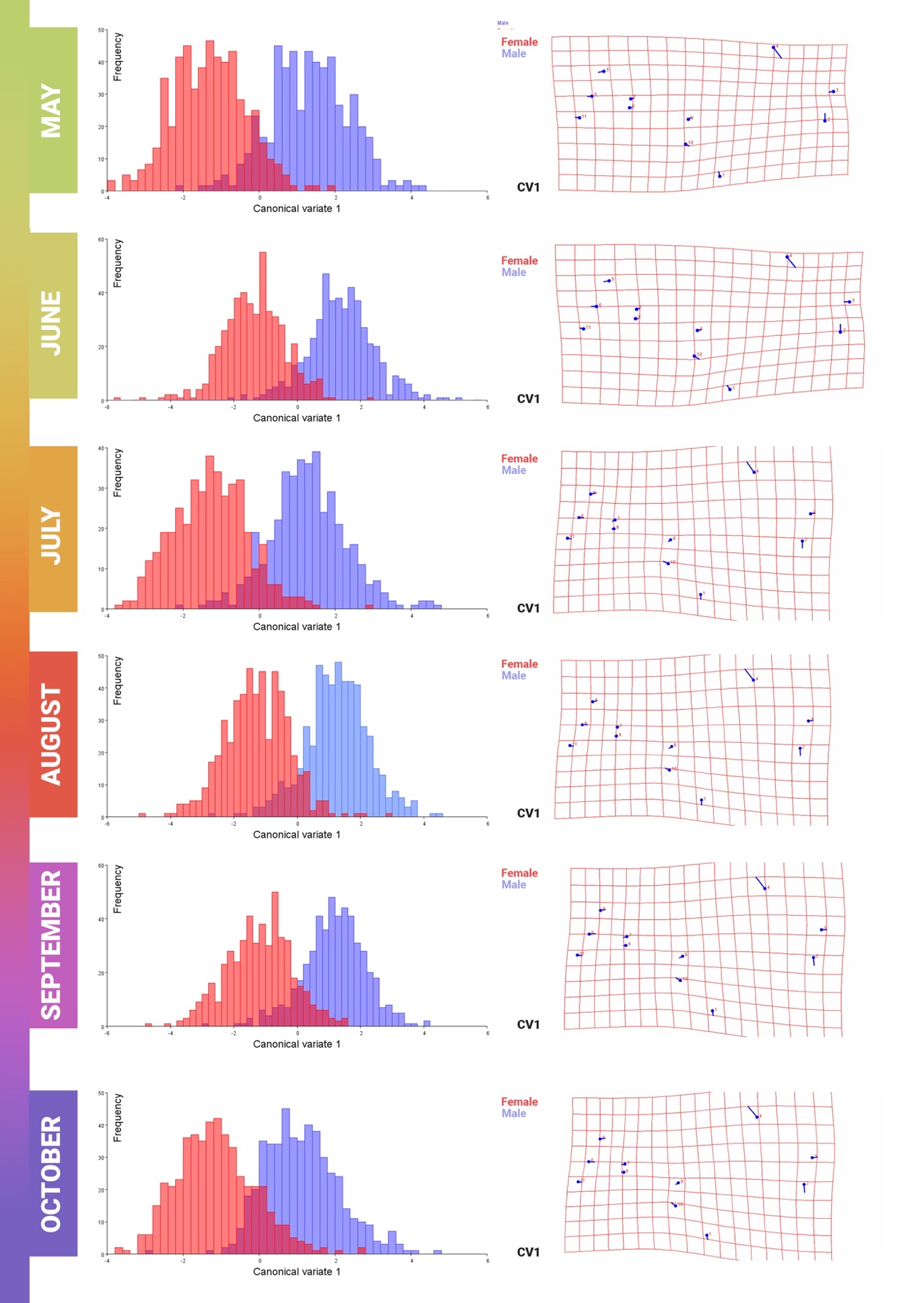
Figure S3. CVA for sexes by month. Left panel CVA analyses, right panel transformation grids.


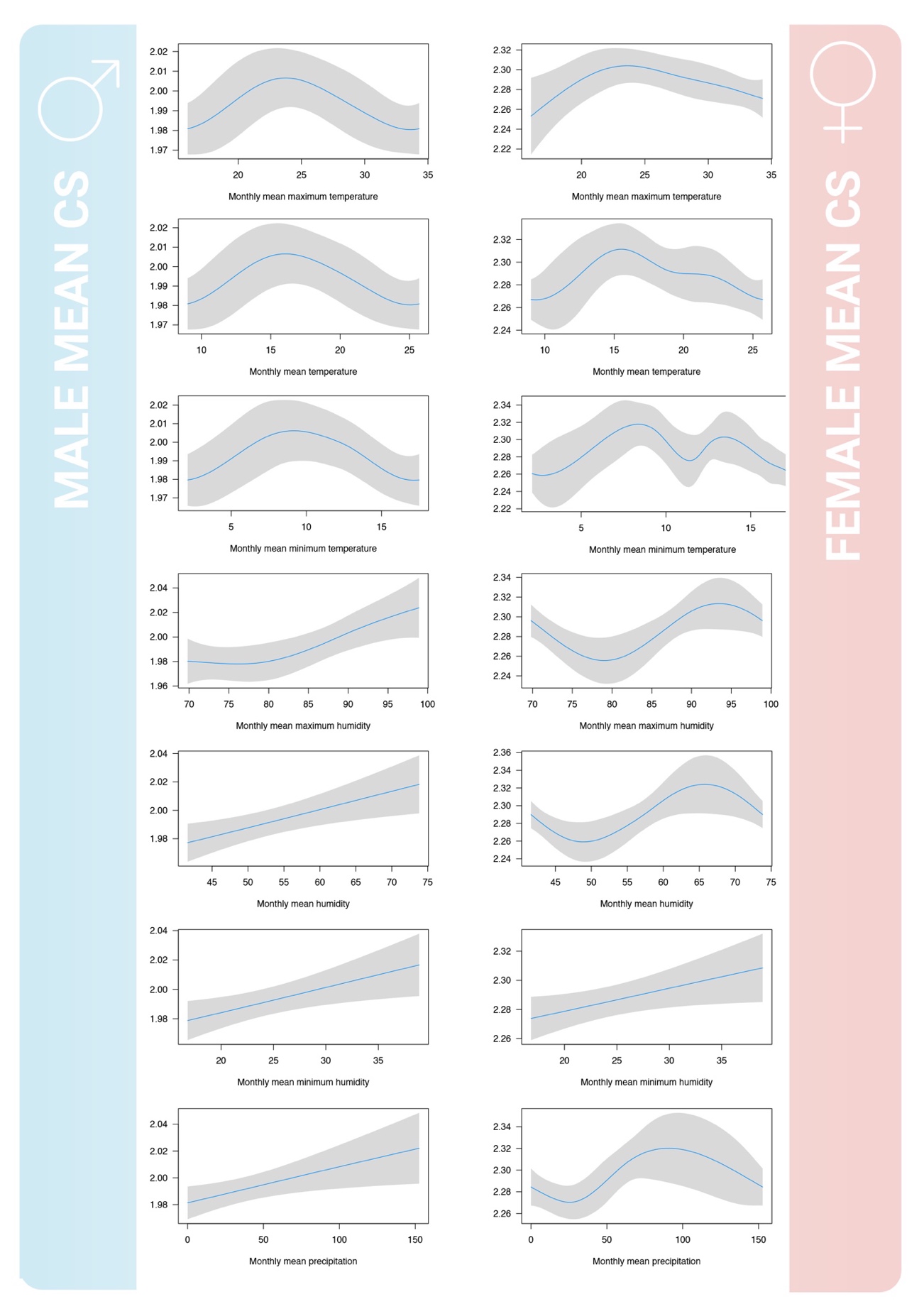


Figure S4. Partial effect of the GAMs model is represented for each relationship between wing size as dependent and climatic variables as independent variable. Lines correspond to the fit of the GAM model. The shaded areas indicate the 95% confidence interval.
